# Assessing caregivers’ perceptions of treatment-seeking for suspected severe malaria in the Democratic Republic of the Congo

**DOI:** 10.1186/s12936-023-04737-6

**Published:** 2023-10-13

**Authors:** Jean Okitawutshu, Antoinette Tshefu, Jean-Claude Kalenga, Giulia Delvento, Christian Burri, Manuel W. Hetzel, Christian Lengeler, Aita Signorell

**Affiliations:** 1 Kinshasa School of Public Health, University of Kinshasa, Kinshasa, Democratic Republic of the Congo; 2 Swiss Tropical and Public Health Institute, Allschwil, Switzerland; 3 University of Basel, Basel, Switzerland

**Keywords:** Severe malaria, Malaria infection, Rectal artesunate, Treatment-seeking, Knowledge, attitude and practice, Democratic Republic of the Congo

## Abstract

**Background:**

Malaria remains a major public health issue in the Democratic Republic of the Congo (DRC), accounting for 44% deaths among outpatient visits in children < 5 years of age, and 22% of facility deaths. Understanding determinants of caregivers’ treatment-seeking patterns and decision-making is crucial in reducing the malaria burden.

**Methods:**

In the frame of the Community Access to Rectal Artesunate for Malaria (CARAMAL) project, cross-sectional household surveys that randomly sampled villages and households were carried-out in three rural DRC health zones prior to the rollout of pre-referral Rectal Artesunate (RAS) and then 9 and 19 months after RAS rollout (post-RAS). Data were captured electronically through face-to-face interviews with the main caregivers of children < 5 years. Capillary blood samples of the children were tested for malaria and anaemia. The main study outcome was whether caregiver “sought treatment outside home” when the child had fever. Multilevel mixed effects logistic regression models using village as random effect and health zone as a fixed effect was performed to assess treatment-seeking predictors.

**Results:**

2439 household interviews were completed (pre-RAS 888 and post-RAS 1551), including 316 and 653 treatment-seeking interviews. Overall, 3499 children < 5 years were tested for malaria and anaemia (pre-RAS 1,315 and post-RAS 2184). Caregiver’s recognition of severe malaria signs was poor, while knowledge of symptoms of uncomplicated malaria seemed high. Despite this, danger signs significantly increased the odds of seeking treatment (aOR = 2.12, 95%CI 1.03–4.38), the same was found for the “least poor” quintile (aOR = 3.01, 95%CI 1.03–8.82), as well as residents of Kingandu (aOR = 2.78, 95%CI 1.01–7.65). “Doing something at home” against fever negatively affected treatment-seeking in both study phases. RAS acceptance was high, at almost 100%. Malaria prevalence was higher post-RAS (45.2%) compared to pre-RAS (34.4%), p = 0.003, but anaemia, although high (≥ 75%), was similar in both study phases (p = 0.92).

**Conclusion:**

In remote communities with high malaria prevalence in the DRC, malaria remains a major problem. Improving the recognition of danger signs of severe disease and introducing pre-referral RAS may improve treatment-seeking and contribute to reducing malaria-related mortality among children—if quality of care can be guaranteed.

**Supplementary Information:**

The online version contains supplementary material available at 10.1186/s12936-023-04737-6.

## Background

Several strategies are being implemented to reduce the unacceptable high burden of malaria in sub-Saharan African countries, where an estimated 228 (95%) million new malaria cases globally occurred in 2020, leading to 602,000 deaths [1]. The Democratic Republic of the Congo (DRC) accounts for 12% of that global burden of malaria [1]. Despite substantial improvements in the prevention and treatment during the past years, malaria remains the principal cause of morbidity and mortality, accounting for 44% deaths among outpatient visits in children under the age of 5, and 22% of facility deaths [2, 3]. Approximately 97% of the Congolese population lives in areas with high and stable malaria transmission, with a transmission period lasting 8 to 12 months per year [2]. The most common vector encountered is *Anopheles gambiae *sensu lato (*s.l.*), and *Plasmodium falciparum* is the most common malaria parasite responsible for the majority of severe cases [2, 4]. The prevalence of malaria varies widely across the country and even within urban areas such as Kinshasa [5]. A great variation was also generally observed between rural and urban areas, and across age groups [6]. The Eastern regions account for the lowest prevalence (< 10%), while in zones situated in the North and the Center of the country it reaches 39% [7, 8].

To align with both international and national guidance, the National Malaria Control Programme (NMCP) of DRC has developed the 2020–2023 strategic plan to guide high-impact interventions to reduce malaria-related morbidity and mortality [3]. The interventions listed include the universal distribution of long-lasting insecticidal nets (LLIN), Intermittent Preventive Treatment in pregnancy (IPTp), the treatment of severe malaria with injectable artesunate, and the treatment of uncomplicated malaria using artemisinin-based combination therapy (ACT) [3]. Despite these efforts, the malaria mortality burden remains high, particularly in children. Hence, new interventions are required, including the pre-referral treatment with Rectal Artesunate (RAS) in children with suspected severe malaria in remote areas. Pre-referral treatment allows to initiate anti-malarial treatment soonest, and provide increased safety for the referral to a higher-level health facility [9]. Since referrals can be lengthy [10, 11], and any intervention to speed up the initiation of malaria treatment could potentially save lives and prevent complications.

RAS has been shown to be a safe and efficacious anti-malarial medicine [12–14]. A multi-country randomized controlled trial including two African countries (Ghana and Tanzania) showed that RAS was efficacious in saving lives and reducing permanent disability, when administered by community volunteers, and when the referral time to a health facility exceeded 6 h (51% protective efficacy) [15]. The acceptability of RAS was universally found to be high among caregivers and community-based health workers [14, 16–18].

The Community Access to Rectal Artesunate for Malaria (CARAMAL) project was designed as a large-scale operational pilot study in three African malaria-endemic settings including the DRC, Nigeria and Uganda, to provide evidence of the real-life public health value of RAS [19]. It also aimed to assess health care seeking patterns [20], severe malaria case management at community and referral facility levels and describing RAS use and acceptance [16, 21, 22]. CARAMAL also assessed real world cost associated with the RAS rollout [23]. Finally, the project intended to advance the development of operational guidance to catalyze effective and appropriate scale-up of RAS as pre-referral treatment for severe malaria.

As part of the CARAMAL study, the present work aimed to (1) determine the prevalence of malaria infection and anaemia in children under 5 years of age at community level in the DRC, (2) measure the level of caregiver’s knowledge and attitudes towards malaria and pre-referral rectal artesunate, and (3) understand determinants of caregiver’s treatment-seeking patterns and decision-making. These are essential elements in understanding the real-world effectiveness of this seemingly simple intervention used in a complex health care system, as well as optimizing its implementation at large scale in settings with limited resources.

## Methods

### Overall study design 

CARAMAL was an observational study based on a before-and-after plausibility design accompanying RAS rollout (April 2019) in DRC, Nigeria and Uganda through established community-based health providers [19]. The patient surveillance system (PSS), the main component of the project evaluation was setup and maintained over the two study phases: the pre-RAS phase lasted 10 months prior to RAS roll-out (from June 2018 to March 2019), while the Post-RAS phase lasted 16 months (from April 2019 to July 2020). The PSS enabled to recruit severely ill children seeking care from a community-based healthcare provider under the age of 5 years based on the presence of iCCM danger signs, and then track them from admission up to 28 days later at home. A cross-sectional household survey to complement the longitudinal PSS findings was run 3 months prior RAS rollout (baseline, January 2019) and then repeated 9 months (midline, January 2020) and 19 months (endline, November 2020) after RAS introduction.

### Study setting

Three repeated cross-sectional surveys were conducted in a sample of villages randomly selected at each survey round within the three CARAMAL study areas in DRC: the Health Zone (HZ) of Kenge in Kwango Province, around 280 km away from Kinshasa, as well as the Health Zones of Ipamu and Kingandu in Kwilu Province, roughly 825 km and 650 km from Kinshasa. The three HZs cover together 933 villages (Kenge 507, Ipamu 206 and Kingandu 220) and had an estimated total population of 785′968 inhabitants, of which 145′107 were children under the age of 5 (https://www.worldpop.org). The health system included a peripheral level of care with 42 community health care sites (CHCS) and 152 primary health care facilities (PHC), as well as a reference level of care consisting of 19 referral health facilities (RHF)—16 referral health centers and 3 general referral hospitals. The three sites have a tropical climate with two seasons: dry season from May–September, and a rainy season from October–April. More details on the CARAMAL study settings and the study design are provided elsewhere [19], while essential impact and implementation results are also available in other publications [20–22, 24].

### Sample size

The sample size for the household surveys was based on a comparison of treatment-seeking rates from community-based providers between baseline and post-implementation of RAS. For comparing proportions pre- and post-RAS implementation, a treatment-seeking rate at baseline was estimated conservatively to be as low as 15%, based on historic reports of treatment-seeking for fever in children < 5 years from formal health facilities of between 15% [25] to over 75% [26]. For detecting an increase of 20% with 80% power and α = 0.05, a minimum sample size of 906 household survey responses on treatment-seeking for severe febrile illness were required per survey round.

### Study participants

The sampling frame consisted of all 933 villages in the three HZ in the CARAMAL project area. A multi-stage random cluster sampling procedure was applied to select households. For the baseline and midline survey rounds, 32 villages (Ipamu 11, Kenge 15 and Kingandu 6; number proportional to the size of the HZ) were randomly sampled from complete village lists. In the endline survey, Ipamu HZ was not included due to logistical constraints, hence only 20 villages were selected. In each sampled village, an exhaustive list of households with at least one child under 5 years was drawn up within the village boundaries, extended to the nearest village when the number of households was less than 30. Global positioning system (GPS) coordinates of the village boundaries were captured by data collectors in collaboration with the head of the village and the CHW. Among the listed households, 30 were randomly selected to participate in the survey.

All members of sampled household were listed and a face-to-face interview about household characteristics was conducted with the household head or another adult household member. Caregivers of children under 5 years took the second section of the interview related to treatment-seeking behaviour. The survey included only household heads and caregivers who had provided written informed consent. Households / caregivers without children < 5 years, those without permanent residence in the project area, and those who did not speak any of the local languages were not eligible for the interview.

### Rectal artesunate rollout

World Health Organization (WHO) pre-qualified RAS suppositories each containing 100 mg of artesunate were deployed in the study areas by UNICEF, in close collaboration with national and local health authorities. Health care providers including Community Health Workers (CHWs), nurses and doctors underwent refresher trainings on the integrated community case management (iCCM) by local health authorities, with support from the NMCP and other programmes implementing iCCM. These trainings focused on the recognition of iCCM general danger signs, the pre-referral treatment of suspected severe malaria with RAS at community level, and appropriate case management at referral health facilities. In parallel, a Behaviour Change Communication (BCC) campaign was launched through local media with key messages on malaria, and on the benefits of seeking health care at the nearest CHW or health facility. Following a 9 months pre-RAS phase, RAS was introduced in almost all CHWs and PHCs. The decision for RAS administration was based on iCCM guidelines [27], and in accordance with the manufacturers’ dosage recommendation: one suppository of 100 mg for children between 6 months to < 3 years, and two suppositories for those from 3 to less than 6 years of age (https://www.mmv.org/research-development/project-portfolio/artecaptm). According to the iCCM algorithms, the decision criteria for a child to be given RAS included fever or a history of fever and the presence of at least one of the following iCCM general danger signs: (1) convulsions, (2) difficulty drinking or feeding, (3) repeated vomiting, and (4) unusually sleepy or unconscious [27] regardless of malaria test result.

### Data collection

#### Procedures

Prior to starting the survey, data collectors underwent three days of extensive training focused on the survey methodology, ethics, data collection tools and procedures. Face-to-face interviews were conducted using a pre-tested electronic structured questionnaire. All data collection tools were programmed as Open Data Kit (ODK, https://opendatakit.org/) forms on android tablet computers.

#### Questionnaires

The structured household questionnaire had two sections: (1) the household information capturing demographics of all household members; age, sex, education and religion of the household head; indicators of the household’s socio-economic status (household ownership of assets); LLIN coverage; household head’s knowledge related to malaria. (2) Treatment-seeking patterns for an episode of fever or history of fever during the 14 days preceding the survey for those households that experienced such an episode. The treatment-seeking interview captured signs and symptoms of the illness and subsequent treatment-seeking patterns. It also captured data related to knowledge and attitudes of the caregivers towards RAS and their experiences with RAS.

### Blood testing

A finger or heel-prick capillary blood sample was collected to test children < 5 years for malaria, using either the CareStart™ Malaria Pf (HRP2) antigen test (Access Bio, Inc., Somerset, New Jersey, USA), or the SD-Bioline *P. falciparum/pf* malaria Rapid Diagnostic Test (RDT; Standards Diagnostics, Kyonggi, Republic of Korea). In addition, haemoglobin (Hb) concentration was measured with a handheld photometer (HemoCue Hb 201 + , Ängelholm, Sweden). Results were recorded in a separate ODK form.

### Study outcomes

The primary outcome of the present study was whether caregivers “sought treatment outside home” (yes/no) when the child had fever with or without danger signs. Predictors of interest included age, sex, education, religion of child’s caregiver, households’ socioeconomic status (SES) expressed as wealth quintiles, presence of iCCM general danger signs, location (Heath Zone), “did something at home” (yes/no) including self-medication, cold bath, damp envelopment and any other practice to kill fever, and “took antimalarial at home” (yes/no). Secondary outcomes included the prevalence of malaria infection (RDT positive/negative), anaemia (Hb < 11 g/dL) and fever (current or history of fever in the past 48 h preceding the survey).

### Data analysis

Field supervisors carried out regular daily checks of the completed forms and uploaded them via internet to the secured ODK Aggregate server at the Swiss Tropical and Public Health Institute (Swiss TPH) in Switzerland. Forms were downloaded as CSV datasets from ODK Aggregate through ODK-Briefcase-v1.13.1, cleaned and analysed in Stata SE V.16.1. (Stata Corporation, College Station, TX, USA) [28]. By combining household and household members’ ownership of assets, livestock ownership and background characteristics of dwellings, a composite household wealth index was computed using principal components analysis (PCA) in STATA to determine households’ SES in five quintiles (poorest, poor, medium, wealthy and wealthiest) [29, 30]. Analysis of treatment-seeking predictors was restricted to households in which the child had a history of fever in the 2 weeks preceding the survey, had already completed the treatment-seeking path, and were no longer sick.

Quantitative data was expressed as means and standard deviation (SD), or as medians and interquartile range (IQR). Means were compared using unpaired t-test or the Wilcoxon rank sum test when t-test validity criteria were not met. Categorical data was summarized as proportions with their 95% confidence intervals (95% CI), and the Pearson Chi-square test or Fisher’s exact test were used to compare them. A p-value of less than 0.05 was considered statistically significant.

Crude odds ratios (OR) with their 95% CI were computed prior to building the final multilevel-mixed effects logistic regression models, using cluster (village) as random effect to adjust for clustering and including health zone as a fixed effect. Results were expressed as adjusted odd ratios (aOR) with their 95% CI. All results were divided into two study phases (pre-RAS versus Post-RAS), except for symptoms and danger signs displayed by children < 5 years, which were only elicited after RAS introduction.

### Weights

Analysis weights during the analysis were calculated as the inverse of a household / an individual’s probability of being selected. The same weights were applied to household level indicators and to those related to children < 5 years within a particular household. The sampling weights were calculated by sampling stage, excluding the level of stratification that was HZ (Additional file 2: Table S1).

### Ethics 

The CARAMAL study protocol was approved by the Research Ethics Review Committee of the World Health Organization (WHO ERC, No. ERC.0003008), the Ethics Committee of the University of Kinshasa School of Public Health (No. 012/2018) and the Scientific and Ethical Review Committee of CHAI (No. 112, 21 Nov 2017). The study was registered on ClinicalTrials.gov (NCT03568344).

A written informed consent was obtained from all household heads prior to starting the interviews. Data collectors disclosed promptly blood test results to the child’s caregiver and advised those whose children tested positive for malaria, and or those with anaemia to attend the nearest health facility for additional assessment and appropriate treatment, in accordance with national policy. Children found with severe anaemia were brought immediately to the nearest health facility by the field teams.

## Results

### Study population

Overall, 84 villages over three rounds were randomly selected from complete lists of villages and surveyed in the three HZ: 32 (baseline), 32 (midline), and 20 (endline). The number of households sampled was 926 during the pre-RAS phase, and 1553 during the post-RAS phase. Households with completed interviews were 888 and 1551, respectively. In total, 969 treatment-seeking interviews were completed with caregivers of children under the age of five: 316 pre-RAS, 653 post-RAS. Of the 3499 recorded children < 5 years from whom blood samples were collected for malaria testing and Hb measurement, 1315 were at pre-RAS and 2184 at post-RAS (numbers shown in Additional file 1: Figure S1).

### Household characteristics

Table 1 displays household heads and household characteristics, by study phase. More than 80% of household heads were 30 years old or more. The majority of household heads were male (88.6%). Overall, 7 out of 10 household heads were of Christian faith (Catholics or other Christians), whereas one third reported being “non-Christian” including Muslims 1.2% (30/2′439), “traditionalist” 4.9% (119/2′439), those practicing an “other religion” 14.4% (353/2′439), and very few reported having no religion at all 7.7% (187/2′349). Concerning the highest level of education achieved, 61.3% of household heads had completed secondary school and above, while almost 10% had no education or did not answer the question. The median number of LLIN owned by household was 2 (IQR 1–3) in a subset of 1′740 households: 740 pre-RAS, 1,000 post-RAS (of which 716 from midline survey 284 from endline survey). However, this proportion appeared to decrease over-time from 3 (2–3) at pre-RAS (83.5%, 95%CI 76.4–88.8) to 2 (1–2) at post-RAS (64.6%, 95%CI 60.4–68.7), p < 0.001. This downward trend resulted from the fact that the last LLIN distribution had taken place in 2018.

**Table 1:** Household heads and household characteristics, by study phase

Characteristics	Pre-RAS N = 888		Post-RAS N = 1551		Pooled N = 2439	
	%	95% CI	%	95% CI	%	95% CI
Age						
15–29	15.6	11.9–20.1	18.2	15.4–21.3	18.1	15.5–21.2
30–39	40.5	35.7–45.6	37.7	34.9–40.7	37.8	34.9–40.6
40–49	43.9	39.7–48.2	44.1	40.5–47.8	44.1	40.5–47.8
Sex						
Male	87.7	84.9–90.1	88.6	86.0–90.8	88.6	86.0–90.7
Female	12.3	9.9–15.1	11.4	9.2–14.0	11.4	9.3–14.0
Religion						
Christians	68.1	62.7–73.1	70.6	65.0–75.7	70.6	65.1–75.6
Non-Christians	31.9	26.9–37.3	29.4	24.3–35.0	29.4	24.4–34.9
Education						
No education / no answer	12.6	9.1–17.4	10.9	9.1–13.0	10.9	9.1–13.0
Primary	21.3	16.9–26.5	27.9	24.4–31.7	27.8	24.4–31.6
Secondary and above	66.1	58.0–73.4	61.2	57.4–64.9	61.3	57.6–64.8
Wealth quintile						
Poorest	26.1	18.2–36.0	21.6	17.8–26.0	21.7	17.9–26.0
Second	13.0	9.8–17.0	16.2	14.1–18.6	16.2	14.1–18.5
Middle	18.7	14.1–24.5	20.7	18.8–22.8	20.7	18.8–22.7
Fourth	18.4	15.7–21.3	19.8	17.3–22.5	19.8	17.4–22.4
Least poor	23.8	11.7–42.4	21.7	16.9–27.4	21.7	17.0–27.3
LLIN ownership	N = 740		N = 1000		N = 1740	
Median (IQR)	3 (2–3)		2 (1–2)		2 (1–3)	

Endline survey conducted in only two out of three health zones (see text)

*N* 2439 households, *LLIN* Long-Lasting Insecticidal Net, *IQR* Interquartile Range

### Prevalence of malaria infection and anaemia among children under the age of 5 years

The proportions of children under 5 years old that had fever (axillary temperature ≥ 37.5 °C) or a history of fever, a malaria infection (RDT positive) and anaemia (Hb < 11 g/dL) are summarized in Table 2. The median (IQR) age was 2 (1–4) years and each sex represented approximately half of the children surveyed. Children recruited in surveys in both study phases were similar in terms of age (rank sum test = 0.78, p = 0.44). History of fever in the last two days preceding the survey was reported in similar proportions of children at pre-RAS: 19.8% (95% CI 16.0–24.4) and post-RAS: 18.8% (95% CI 15.3–22.9), p = 0.71.

**Table 2:** Prevalence of fever, malaria infection and anaemia among children under 5 years

Indicator	Pre-RAS N = 1315		Post-RAS N = 2184		Pooled N = 3499		*P-value comparing Pre-RAS and Post-RAS*
	%	95% CI	%	95% CI	%	95% CI	
Proportion of children < 5 years with fever during the last 2 days preceding the survey	19.8	16.0–24.4	18.8	15.2–22.9	18.8	15.3–22.8	0.71
Proportion of children < 5 years with current fever (axillary temperature ≥ 37.5 °C)	4.0	3.0–5.3	6.7	5.4–8.3	6.7	5.4–8.2	0.004
Proportion of children < 5 years tested positive for malaria by mRDT	34.4	30.1–39.0	45.2	39.8–50.7	45.1	39.8–50.4	0.003
Proportion of children < 5 years with anaemia (Hb < 11 g/dL)	79.8	74.0–84.5	79.5	77.0–81.7	79.5	77.1–81.7	0.92

Endline survey conducted in only two out of three health zones

*N* = 3499 children, *95% CI* 95% Confidence Interval, *IQR* Interquartile Range, *mRDT* malaria Rapid Diagnostic Test, *Hb* Haemoglobin, *g/dL* gram per deciliter

An increased temperature by axillary measurement was found in fewer cases during the pre-RAS period (4.0%, 95% CI 3.0–5.3), compared to post-RAS (6.7%, 95% CI 5.4–8.3) (p = 0.004). The prevalence of malaria infection was 34.4% (95% CI 30.1–39.0) during pre-RAS, while a significant higher positivity rate of RDT was observed during the post-RAS phase: 45.2% (95% CI 39.8–50.7) (p = 0.003). The prevalence of malaria was heterogeneous in the three health zone where surveys conducted; Kenge HZ had a significantly higher overall prevalence of malaria (66.5%) compared to Kingandu HZ (26.3%) and Ipamu HZ (7.2%), p < 0.001. These observed difference in the prevalence of malaria pre-post RAS might be in part be explained by the missing Ipamu Health Zone in the endline survey (Ipamu had generally a lower prevalence rate), and the lower coverage of LLINs (Table 1). In addition, seasonality might also reduce comparability, since the endline survey was carried out in November, corresponding to the “high transmission season”, while the baseline survey was carried out in late January and early February, during the moderate transmission period. Care should therefore be taken in interpreting the trend in the prevalence data.

A slightly higher rate of anaemia (Hb < 11 g/dL) was observed pre-RAS (79.8%, 95% CI 74.0–84.5) compared to post-RAS (74.5%, 95% CI 77.0–81.7), but the difference was not statistically significant. When looking at anaemia by HZ, a similar trend than for malaria infection was observed: the highest rate was in Kenge HZ (57.5%) followed by Kingandu HZ (30.3%) and Ipamu (12.2%). The mean value of haemoglobin in this young population was 9.7 (± 1.6) g/dL. There was no evidence of difference in mean Hb levels between children enrolled in both study phases: pre-RAS [9.7 (± 1.7) g/dL] versus post-RAS [9.7 (± 1.6) g/dL]; t-test = 0.94, p = 0.35).

## Knowledge and attitudes towards malaria and RAS

### Knowledge of malaria, RDT, ACT and RAS

**Fig. 1:**
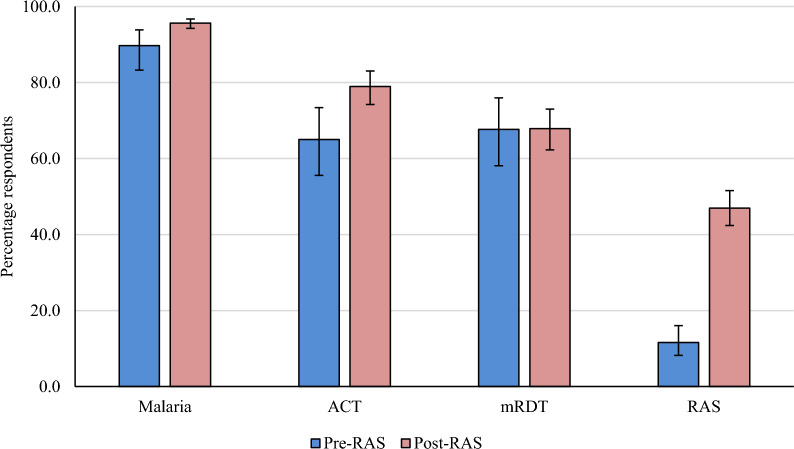
Percentage of parents/household head declaring having heard of malaria, mRDT, ACT and RAS, by study phase. N = 2439 (pre-RAS, N = 888, post-RAS N = 1551). *mRDT* malaria Rapid Diagnostic Test. *ACT* Artemisinin-based Combination Therapy. *RAS* Rectal artesunate

Figure 1 displays the proportion of caregivers who declared having heard of malaria, RDT, ACT and RAS, by study phase. Overall, the percentage of caregivers that reported having heard of malaria was high, and it increased significantly over time [89.7% (95%CI 83.3–93.9) at pre-RAS; 95.6% (95%CI 94.2–96.7) at post-RAS, p = 0.004]. RDT is one of the most common malaria diagnostic tools known by people seeking care at both community and health facility level in rural areas. The proportion of caregivers that reported having heard of RDTs was almost 68% in both study phases (p = 0.97). In the pre-RAS phase, 65.0% (95%CI 55.6–73.4) of caregivers mentioned they had heard of ACT, and this proportion increased significantly by almost 14 percent points post-RAS implementation (p = 0.004). RAS was newly introduced in the study areas, and this certainly explained the low proportion of caregivers reporting having heard about it during the pre-RAS survey (11.6%, 95%CI 8.2–16.1), while this proportion raised significantly (p < 0.001) during the post-RAS surveys (47.0%, 95%CI 42.4–51.6).

### Reported symptoms of malaria by caregiver of children < 5 years

**Fig. 2:**
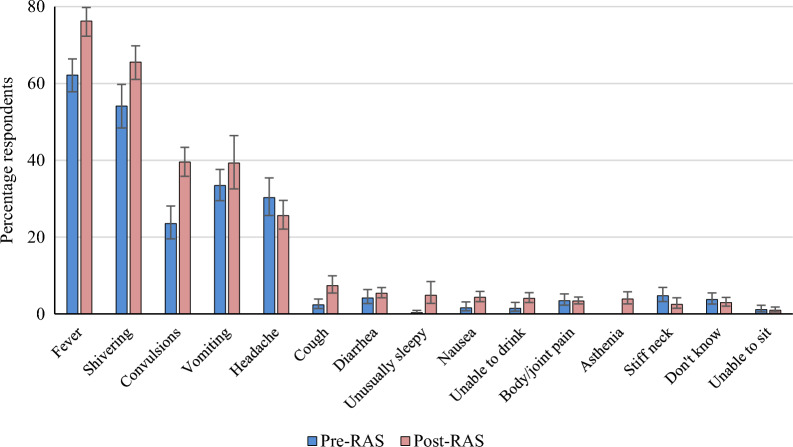
Mentioned symptoms of malaria by parents/caregivers of children < 5 years, by study phase. N = 2439 (pre-RAS, N = 888, post-RAS N = 1551)

The knowledge of caregivers related to symptoms of malaria was assessed by asking them to list all symptoms of malaria they were aware of (Fig. 2).

As might be expected, “fever” was the leading malaria symptom in all survey rounds: between 62.2% (95%CI 57.8–66.4) and 76.3% (95%CI 72.3–79.8), p < 0.001. This was followed by “shivering”: 54.2% (95%CI 48.4–59.8) at pre-RAS and 65.7% (95%CI 61.1–69.8) post-RAS (p = 0.002). Convulsion, which is an important danger sign for severe malaria was reported by only 23.5% (95%CI 19.5–28.1) of respondents at pre-RAS and had significantly increased in the post-RAS phase (39.6%, 95%CI 35.9–43.4), p < 0.001. Although community sensitization highlighted two other iCCM general danger signs including “unusually sleepy” and “unable to drink”, only few caregivers cited the first (0.3% pre-RAS versus 4.9% post-RAS), while the second ranged from 1.4% pre-RAS survey to 4.1% post-RAS.”Vomiting” and”headache” were mentioned by one third of respondents at baseline and this increased slightly at endline for “vomiting” (39.3%), while the reverse was observed for “headache” (25.6% at post-RAS). Other common symptoms such as “diarrhoea”, “nausea”, “body/joint pain” and “asthenia” were reported by less than 6% of caregivers. Reported symptoms that are not diagnostic for malaria included respiratory symptoms, such as “cough”, and one sign typical for meningitis: “stiff neck”.

### Reported treatments for children with malaria

**Fig. 3:**
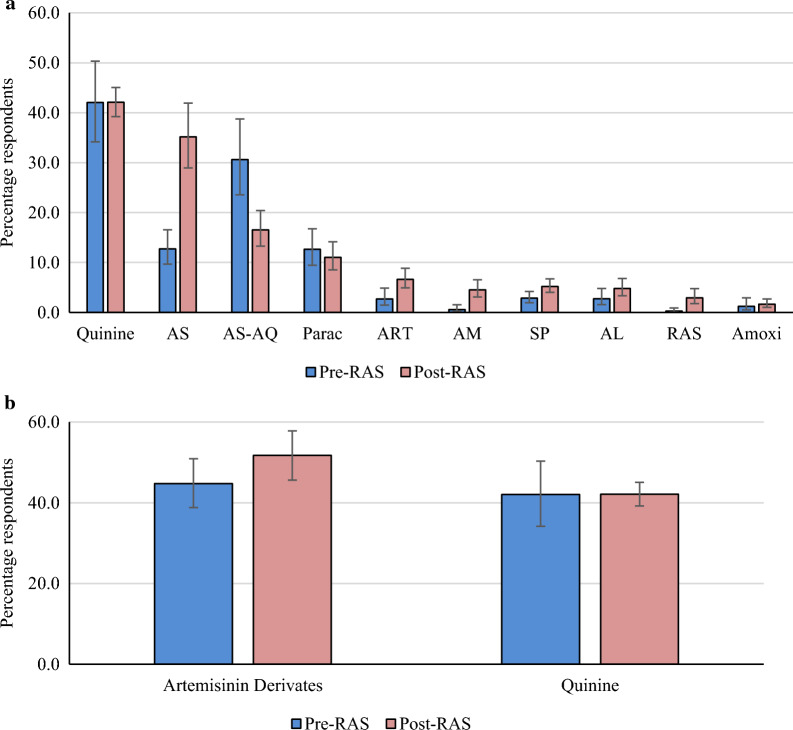
**a** Mentioned treatment of child with malaria, by study phase. N = 2439 (pre-RAS, N = 888, post-RAS N = 1551). AL = Artemether-Lumefantrine. AM = Amodiaquine. Amoxi = Amoxicillin. ART = Artemether. AS = Artesunate. AS-AQ = Artesunate-Amodiaquine. Parac = Paracetamol. Quinine = Injectable and oral quinine. RAS = Rectal Artesunate. SP = Sulfadoxine/Pyrimethamine. **b** Mentioned treatment of child with malaria, comparing proportions of Artemisinin derivates and quinine, by study phase. N = 2439 (pre-RAS, N = 888, post-RAS N = 1551). Artemisinin derivates include injectable (artesunate, artemether) and oral (Amodiaquine, AS-AQ and AL). Quinine = Injectable and oral quinine

Caregivers were also asked to list all treatments of malaria they were aware of (Fig. 3a). Quinine (injectable and oral) was the most frequently mentioned treatment (42.0%) in both phases of the study, followed by artesunate (12.7%, 95%CI 9.7–16.6) at pre-RAS, which significantly increased to 35.2% (95%CI 29.0–41.9) post-RAS, p < 0.001. Artesunate-amodiaquine (AS-AQ) went the opposite way: 30.6% (95%CI 23.5–38.7) at pre-RAS and 16.5% (95%CI 13.3–20.4) at post-RAS, p < 0.001. Sulfadoxine/pyrimethamine (SP) was reported by only 2.8% and 5.2% during the pre-RAS and post-RAS phases, respectively, p = 0.010. RAS was mentioned by only 0.2% or respondents during the pre-RAS survey, when it was basically not available; it significantly rose to 2.9% post-RAS (p < 0.001). This was an unexpectedly low proportion, but since RAS is only given to small children with danger signs, maybe this should have been expected. Even though amoxicillin is an antibiotic and not an antimalarial, 1.2% and 1.7% of respondents mentioned it as a malaria treatment during the pre-RAS and post-RAS phases, respectively.

### Reported symptoms and danger signs reported by caregiver when RAS was given to their child (post-RAS phase)

**Fig. 4:**
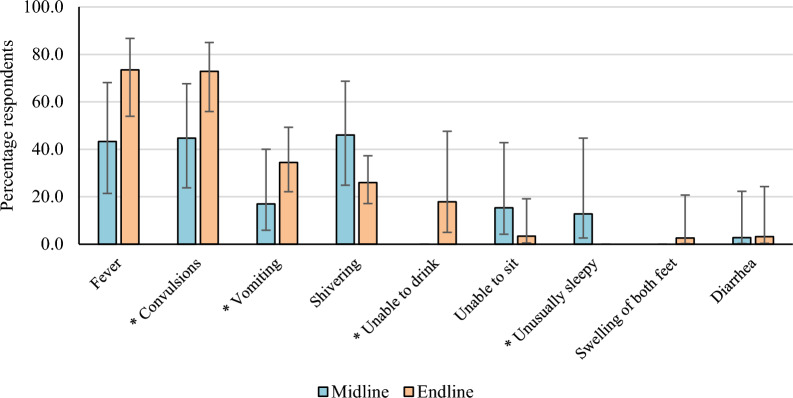
Symptoms and danger signs reported by caregiver in children < 5 years when RAS was given, post-RAS phase. N = 48 (Midline N = 19, Endline N = 29). *Danger sign according to iCCM guidelines

During both post-RAS survey rounds, a low proportion of participants (8% at each survey, N = 665) reported that their children were given RAS by a community-based provider. Symptoms and danger signs reported for children < 5 years receiving RAS are shown in Fig. 4. Fever and convulsions were the most cited: 43.3% (95%CI 21.4–68.1) and 44.7% (95%CI 23.8–67.7) at midline, versus 73.5% (95%CI 54.0–86.8) and 72.9% (95%CI 56.0–85.0) at endline (p = 0.05). “Unable to sit” was cited for 15.3% (95%CI 4.2–42.8) of the cases at midline and only 3.4% (95%CI 0.5–19.1) at endline, while swelling of both feet was only mentioned for 2.6% of cases at endline.

While reports of vomiting significantly increased between mid- and endline (17.0% versus 34.5%), the trend was inversed for shivering (46.0% versus 25.9%). “Unable to drink” was mentioned by only 17.9% (95%CI 4.9–47.6) of respondents at endline, while “unusually sleepy” was even less cited: only 12.8%, (95%CI 2.6–44.7) at midline.

### Source of RAS per caregivers’ perspective (post-RAS phase)

When asked about the source of RAS given to their children, Primary Health Centers (PHCs) were reported most frequently during both survey rounds [90.6% (16/19) midline, 68.1% (17/28) endline], while provision of RAS by CHWs increased from 9.4% (3/19) at midline to 29.5% (10/28) at endline. Lastly, 93.3% (17/19) and 100.0% (28/28) of caregivers interviewed during the mid- and endline survey, respectively, declared that they would want RAS again for their child in case of illness.

### Predictors of treatment-seeking outside home

In a final analysis potential predictors related to caregivers’ treatment-seeking patterns and decision-making for seeking treatment outside the home for their children < 5 years with fever, were assessed before and after RAS rollout. Only the subset of those whose children had a history of fever in the fourteen days prior the survey was analysed (Table 3): pre-RAS 316 caregivers, merged midline and endline surveys 653 caregivers. Since caregivers were often women, this explained why the population presented in Table 3 was predominantly female and younger compared to the household heads shown in Table 1. No evidence of association with seeking treatment outside home was found for age, sex, education, religion, duration of fever and taking anti-malarials at home. Similarly, the age of the child did not show any significant association with treatment-seeking, in both a univariate and a multivariate analysis.

**Table 3:** Predictors of treatment seeking outside home for children < 5 years with fever, before and after RAS roll-out

Survey round	Pre-RAS (N = 316)				Post-RAS (N = 653)			
Predictor	%	aOR	95% CI	P-value	%	aOR	95% CI	P-value
Age of caregiver (years)								
15—29	41.1	–	–	–	31.1	Ref.		
30—39	37.0	–	–	–	29.2	0.90	0.49–1.69	0.75
≥ 40	31.4	–	–	–	28.6	1.04	0.56–1.93	0.89
Sex								
Male	31.5	Ref.			27.1	Ref.		
Female	39.0	1.03	0.48–2.20	0.95	32.3	1.36	0.88–2.12	0.17
Education								
No education	40.5	Ref.			33.4	Ref		
Primary	41.9	0.95	0.37–2.44	0.92	25.1	0.76	0.36–1.62	0.48
Secondary and above	35.7	0.58	0.26–1.30	0.19	30.4	0.83	0.41–1.70	0.61
Religion								
Christians	35.8	–	–	–	27.9	Ref.		
Non-Christians	40.8	–	–	–	32.6	1.59	0.96–2.64	0.07
Wealth quintile								
Poorest	37.8	Ref.			23.2	Ref.		
Second	49.2	1.92	0.76–4.84	0.17	27.5	0.54	0.27–1.09	0.09
Middle	44.5	1.95	0.77–4.92	0.16	33.4	0.85	0.44–1.65	0.63
Fourth	28.3	1.03	0.39–2.72	0.95	24.9	0.57	0.29–1.14	0.11
Least poor	33.4	3.01	1.03–8.82	0.045	35.6	0.68	0.34–1.37	0.28
Health zone								
Ipamu	28.1	Ref.			21.2	Ref.		
Kenge	34.8	1.18	0.39–3.55	0.77	25.2	1.09	0.45–2.64	0.84
Kingandu	53.0	2.09	0.52–8.38	0.30	41.4	2.78	1.01–7.65	0.047
Duration of fever								
≤ 2 days	36.0	–	–	–	21.6	Ref.		
3—4 days	35.9	–	–	–	34.4	1.42	0.83–2.44	0.20
≥ 5 days	41.2	–	–	–	28.8	1.29	0.73–2.28	0.39
Danger signs								
No	33.9	Ref.			27.6	Ref.		
Yes	53.7	2.12	1.03–4.38	0.042	36.9	1.52	0.89–2.61	0.13
Did something at home								
No	66.1	Ref.			40.6	Ref.		
Yes	26.2	0.24	0.12–0.46	< 0.001	25.2	0.51	0.32–0.81	0.005
Took antimalarials at home								
No	41.7	Ref.			30.6	Ref.		
Yes	18.2	0.56	0.23–1.37	0.21	21.0	0.94	0.47–1.91	0.87

*N* 316 versus 653 caregivers (pre-RAS versus post-RAS)**,** *aOR* = Adjusted Odds Ratio, *95% CI* 95% Confidence Intervals

In the pre-RAS results, when compared to the poorest quintile as the reference group, the second, middle and fourth quintile of the wealth index showed some association with seeking treatment outside home, but this was not statistically significant. However, caregivers in the “least poor” quintile were more likely to search treatment outside home (aOR = 3.01, 95% CI 1.03–8.82, p = 0.045). No such evidence of association with treatment-seeking was observed during the post-RAS phase, when the behaviour within all quintiles seemed homogeneous and the aORs were actually below 1.

The odds of seeking treatment outside home appeared to increase—but not significantly so—at pre-RAS for Kenge and Kingandu HZ compared to Ipamu as the reference location. At post-RAS, Kingandu residents showed an increased odds of seeking treatment outside home when their children had fever compared to those of Ipamu (aOR = 2.78, 95% CI 1.01–7.65, p = 0.047). The presence of iCCM danger signs increased significantly the odds of seeking treatment outside at pre-RAS in the un-adjusted analysis (aOR = 2.12, 95% CI 1.03–4.38, p = 0.042), but this trend was not statistically significant any more in the adjusted analysis (aOR = 1.52, 95% CI 0.89–2.61, p = 0.13). “Doing something at home” against fever was significantly associated with a decrease in odds of seeking treatment outside home at pre-RAS (aOR = 0.24, 95% CI 0.12–0.46, p < 0.001) and at post-RAS as well (aOR = 0.51, 95% CI 0.32–0.81, p = 0.005).

## Discussion

The purpose of the reported study was to understand the role played by key determinants on the caregiver’s treatment-seeking patterns and decision-making for seeking treatment outside home for children < 5 years with fever. The study also measured caregiver’s level of knowledge and attitudes towards malaria and pre-referral RAS in the context of high prevalence of malaria and anaemia [7, 8].

This study had some of the limitations of observational study designs. Firstly, by restricting analysis of treatment-seeking predictors to households that met certain criteria, children that were still sick were excluded, biasing potentially the assessment towards slightly less serious cases. But since the large majority of fever cases had resolved within two weeks, this bias is likely to have been minimal. Secondly, questions on symptoms and malaria treatment did not distinguish between uncomplicated and severe malaria. Obviously, mild cases were the great majority of those reported, hence the study’s ability to describe treatment-seeking for severe cases was limited. The purpose was more to get a general sense of treatment-seeking and not specifically for severe cases, which was investigated in much more detail through the Patient Surveillance System set-up by CARAMAL [24]. Thirdly, anaemia was defined as Hb < 11 g/dL in a population relatively anaemic leading to a high prevalence of anaemia (almost 80% % in both phases of the study). An additional threshold of Hb < 8 g/dL was added to make findings from this study more comparable with those from other DRC studies. At this cut off point (Hb < 8 g/dL), overall anaemia was 13.6%. Fourthly, the RDT used to test for malaria detected only *P. falciparum*. Since this species represents over 93% of malaria cases in this region [31]*,* the missed cases of other *Plasmodium* species would have been of marginal importance, and the symptoms of non-*falciparum* species would be anyway very similar.

Interestingly, available survey data from both study phases show that most caregivers visited the formal health facilities including PHCs and RHFs, while CHWs were not visited at all, probably due to their overall small number: only 42 across the three study HZ (Additional file 1: Figure S2). This was different to the other two settings of the CARAMAL study [19].

In this study, caregivers’ recognition of danger signs of severe malaria is still problematic. Although their presence indicates a life threatening situation [27], convulsions and vomiting were the only frequently cited danger signs, and these by only 40% of the caregivers in post-RAS surveys. Although they are often present in severe malaria episodes, “unusually sleepy” and “unable to drink” were not well known despite the awareness campaigns surrounding RAS roll-out. The lack of knowledge remains a challenge that need to be addressed in order to improve treatment-seeking for severely ill children at community-based health providers.

Caregivers’ knowledge of malaria, RDT, ACT, and common uncomplicated malaria symptoms (fever and shivering) appeared high and was close to findings published elsewhere [32–34], but not in line with another study from the DRC that found lower levels of knowledge [35]. Such difference can of course arise in a large country such as the DRC. Community sensitization campaigns do not seem to have resulted in malaria related knowledge increasing from pre-RAs to post-RAS, and it is therefore recommended to increase behaviour change and communication campaigns [26]. Different channels could be used to ensure an effective delivery of the message to a broad audience, of which nearly one third attended only primary school or had no education at all. These channels may include media, the churches whose influence on malaria control practices has been demonstrated elsewhere [36]. Trained CHWs might also help spreading message during their home visits, this was already proven by other studies [37, 38]. Caregivers’ knowledge of malaria treatment showed that artemisinin derivatives including injectable artesunate, artemether and ACT were the most cited compared to quinine (injectable and oral). However, quinine is a well-known antimalarial, even manufactured locally in eastern DRC and often used by nearly one third caregivers at home in early onset of malaria symptoms [39]. As a result, the use of quinine is very high (see Fig. 3a) considering that its use is no longer recommended by WHO and the DRC NMCP [9, 40].RAS was newly introduced in the study areas and hence it was not expected to be used in the pre-RAS phase. The little reported use might, therefore, have been due to the confusion with other suppositories or other traditional drugs or preparations commonly given to children under the age of five through the rectal route.

Only roughly one-third of sick children were brought to care outside home within 48 h of onset of symptoms, which is similar to other results from DRC in which where 42% of responders used a formal health facility [35]. This low care-seeking reflects possibly the restricted access to health facilities due to the long distances involved, and also the high costs involved in malaria episodes [41]. In a population in which 69.7% lives under the poverty threshold of US$ 2.15 a day [42], this is not surprising. As might be expected, households classified in the “least poor” quintile were more likely to seek treatment outside home when their children had fever compared to the “poorest” quintile at baseline. This finding is consistent with those published elsewhere [41, 43]. Since SES is known to impact health care access, this was not obvious for other quintiles, which appear to be rather homogeneous due to a population structure that is also homogeneous and predominantly poor [42].

Although the HZ of Kingandu has a poor health facility coverage and often long distances between villages and health facilities [22], the stability and quality of the leadership of local health authorities could have played a key role in the significant increase of caregivers seeking treatment for their children after RAS roll-out, compared to the other two HZ.

“Doing something at home” including self-medication, cold baths and dressing and any other practices to reduce fever declined significantly the odds of seeking treatment outside home. This finding is consistent with evidence from a review of treatment-seeking for malaria [44]. With regard to the value of education, a study conducted in Zambia found a significant association between health-seeking behaviour and level of education (aOR = 1.47, 95% CI 1.13–1.92) [45], which is different to the results from this study. Another study from the DRC, however found a great proportion of participants with recommended behaviour among those more educated (high school or more) [31]. There are no good explanations as to why this study did not observe such an association.

Given the low CHWs coverage in the study areas, RAS experience of caregivers was gathered from a limited number of them (N = 48). PHC facilities were the principal source of RAS and its acceptance was high, similarly to what was found earlier in DRC [17]. Pre-RAS and post-RAS proportions of children with a history of fever (19.8% and 18.8%) was close to findings from the DRC Multiple Indicator Cluster Survey (MICS) 2018 [8] and other surveys [5, 46], but it was lower than in the second DRC Demographic and Health Survey: 29.5% [7].

This study found a high malaria prevalence at both study phases: pre-RAS 34.4% and post-RAS 45.2%. The prevalence of anaemia was also high, at almost 80% in both phases of the study. Hence, malaria remains a huge burden in these hard-to-reach settings, calling for high-impact interventions for both prevention and treatment.

## Conclusion

Despite its limitations, this study highlighted the challenges related to the recognition of danger signs by caregivers of children < 5 years, as well as the related treatment-seeking patterns in one moderate and two high malaria prevalence settings in the DRC. Findings suggest that despite a fairly good knowledge of mild malaria symptoms and treatment, severe malaria signs (danger signs) are rather less well-known. Incorrect knowledge of symptoms of malaria was also common. This was surprising given the awareness campaigns run by the CARAMAL project. Factors such as belonging to the “least poor wealth” quintile, occurrence of danger signs (when recognized), and good leadership of health system operational units (HZ) appeared to promote treatment-seeking. In remote communities with high malaria prevalence in DRC, malaria and especially severe malaria remains a problem that needs to be addressed timely. Popularizing the recognition of danger signs of severe disease through adequate channels may enhance caregiver’s decision making for treatment-seeking and contribute to reducing malaria-related mortality among children—provided the quality of care can be guaranteed.

### Supplementary Information

**Additional file 1: Figure S1.** Sampling flow-charts. **Figure S2.** Main sources of outside treatment visited by caregivers in baseline and midline surveys. Baseline (N = 97). Midline (N = 91). CHW = Community Health Worker. PHC = Primary health care facilities. RHF = Referral Health facilities.

**Additional file 2: Table S1.** Formula used to compute the weights.

## Data Availability

The datasets used and /or analysed in the current study are available from the corresponding author upon reasonable request.

## Abbreviations

95%CI: 95% confidence intervals
ACT: Artemisinin-based combination therapies
AL: Artemether-Lumefantrine
AM: Amodiaquine
aOR: Adjusted odds ratios
ART: Artemether
AS: Artesunate
AS-AQ: Artesunate-amodiaquine
BCC: Behaviour change communication
CARAMAL: Community access to rectal artesunate for malaria
CHCS: Community health care site
CHW: Community health worker
DHS: Demographic and Health Survey
DRC: The Democratic Republic of the Congo
GPS: Global positioning system
Hb: Haemoglobin
HRP2: *Plasmodium falciparum* Antigen Histidine-rich Protein 2
HZ: Health zone
iCCM: Integrated community case management
IQR: Interquartile range
LLIN: Long lasting insecticidal nets
MICS: Multiple indicator cluster survey
MoH: Ministry of Health
RDT: Malaria rapid diagnostic test
NMCP: National Malaria Control Program
ODK: Open data kit
OR: Odds ratio
PCA: Principal components analysis
PHC: Primary health care facility
RAS: Rectal artesunate
RHF: Referral health facility
SD: Standard deviation
SES: Socioeconomic status
SP: Sulfadoxine/pyrimethamine
UNICEF: The United Nations Children’s Fund
WHO: World Health Organization
