# Supplementary material for: Assessing caregivers’ perceptions of treatment-seeking for suspected severe malaria in the Democratic Republic of the Congo
Source: Malar J. 2023 Oct 13;22:308. doi: 10.1186/s12936-023-04737-6 (PMC10571465; doi:10.1186/s12936-023-04737-6)

Additional file S1 to S2

Sampling flow-charts

*Same Health Zones (where the CARAMAL project was implemented in DRC).

Pre-RAS Survey

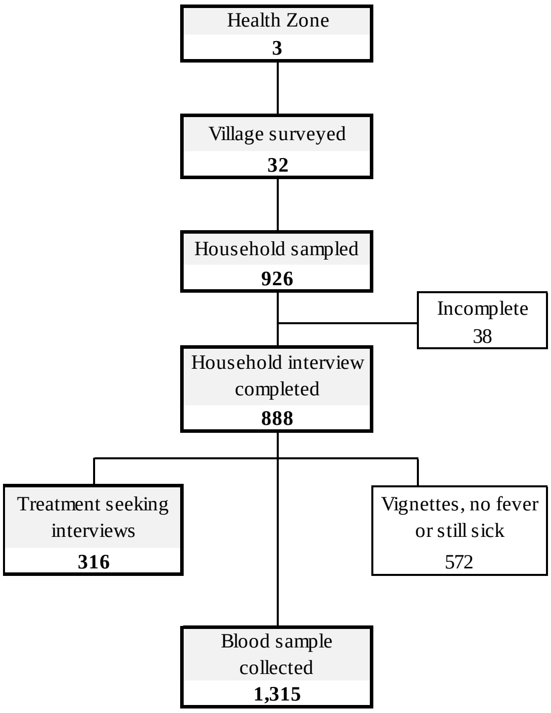

Post-RAS surveys

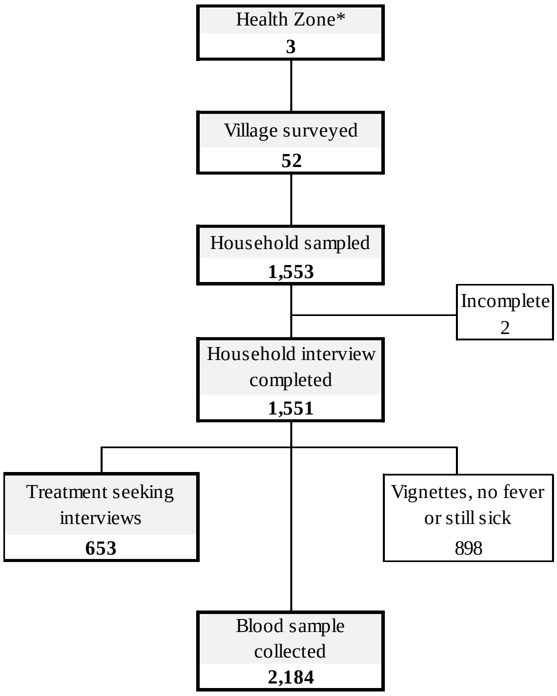

Pooled

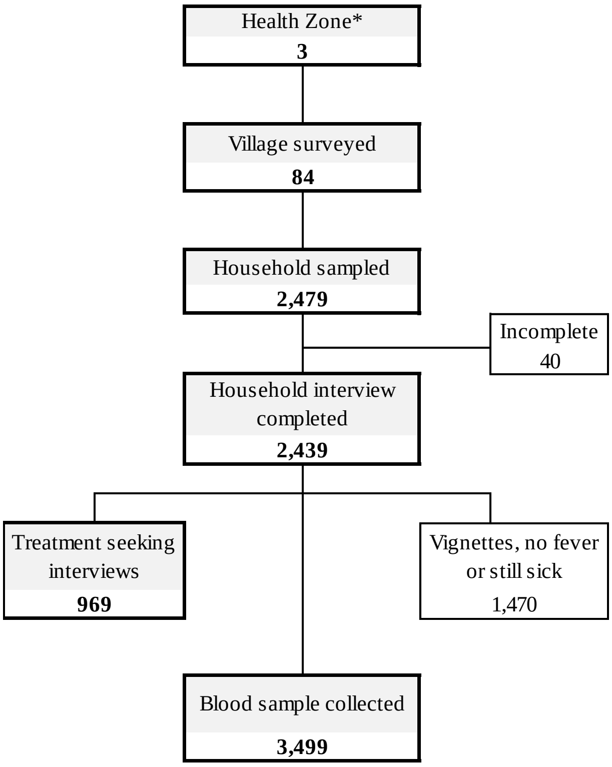

Figure S2: **Main sources of outside treatment visited by caregivers in baseline and midline surveys.** Baseline (N = 97). Midline (N = 91). CHW = Community Health Worker. PHC = Primary health care facilities. RHF = Referral Health facilities.

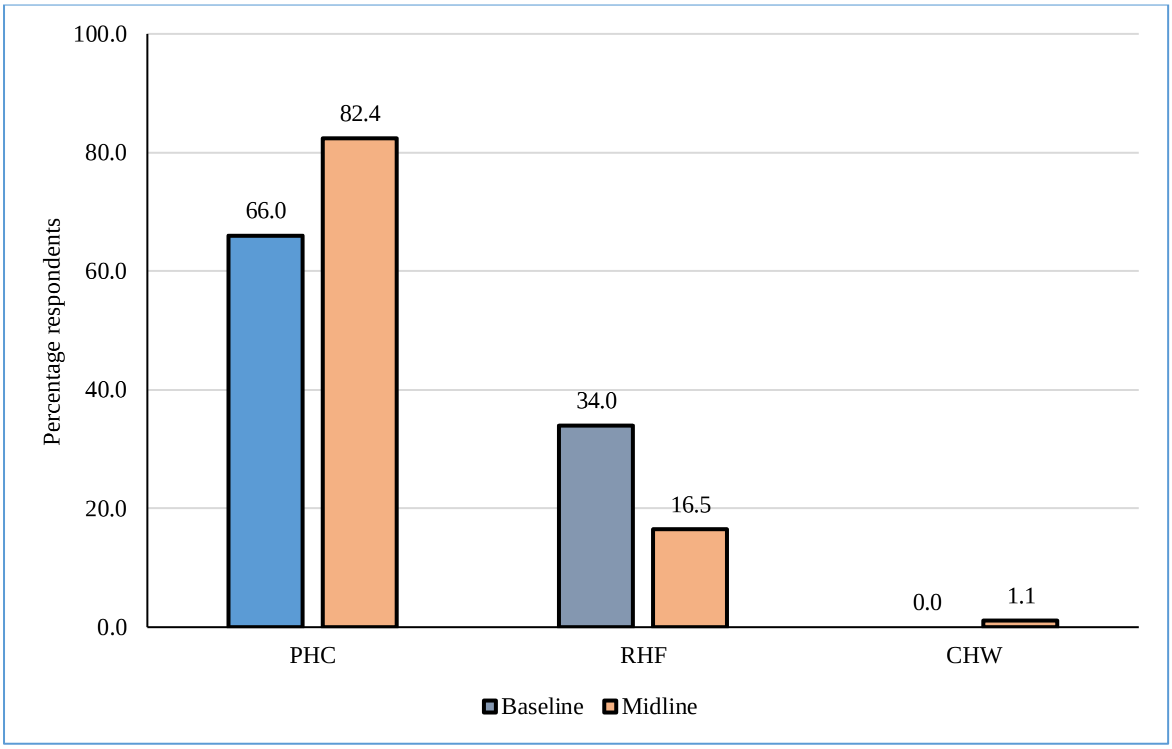

Supplement: Supplementary file 1 — Additional file 1: Figure S1. Sampling flow-charts. Figure S2. Main sources of outside treatment visited by caregivers in baseline and midline surveys. Baseline (N = 97). Midline (N = 91). CHW = Community Health Worker. PHC = Primary health care facilities. RHF = Referral Health facilities. [file 12936_2023_4737_MOESM1_ESM.pdf]
