# Supplementary material for: Assessing caregivers’ perceptions of treatment-seeking for suspected severe malaria in the Democratic Republic of the Congo
Source: Malar J. 2023 Oct 13;22:308. doi: 10.1186/s12936-023-04737-6 (PMC10571465; doi:10.1186/s12936-023-04737-6)
Supplement: Supplementary file 2 — Additional file 2: Table S1. Formula used to compute the weights. [file 12936_2023_4737_MOESM2_ESM.pdf]

Additional file

Table S1: **Formula used to compute the weights**

Weights are related to the probability of a village or a household being selected and were therefore used in the analysis.

$\boldsymbol{pweight_{overall}} = \frac{\mathbf{1}}{\boldsymbol{probability\ of\ village\ being\ selected}} \mathbf{x} \frac{\mathbf{1}}{\boldsymbol{probability\ of\ household\ being\ selected}}$
Whereas:<br>1/probability of village being selected = $\frac{Total\ N\ villages\ in\ stratum}{N\ villages\ selected}$<br>1/probability of household being selected = $\frac{Total\ N\ households\ in\ village}{N\ households\ interviewed}$
